# The convergence of senescence and nutrient sensing during lymphocyte ageing

**DOI:** 10.1111/cei.12876

**Published:** 2016-10-28

**Authors:** A. N. Akbar

**Affiliations:** ^1^ Division of Infection and ImmunityUniversity College LondonLondonUK

**Keywords:** aging, cell differentiation, inhibitory/activating receptors, natural killer cells, T cells

## Abstract

Immunosurveillance requires the migration of lymphocytes and their activation to induce proliferation and effector function. Effective immunity requires an optimal supply of nutrients to lymphocytes. Cells contain nutrient sensing apparatus such as adenosine 5′‐monophosphate‐activated protein kinase (AMPK) that surveys intracellular ATP levels. Immunity declines during ageing and one possibility is that the energy balance may be altered in old lymphocytes. This paper summarizes recent data identifying a convergence of senescence and nutrient signalling pathways in lymphocytes that inhibit both T cell and natural killer (NK) cell function during ageing. Significantly, these pathways can be inhibited to enhance the activity of these cells.

## OTHER ARTICLES PUBLISHED IN THIS REVIEW SERIES

Immunosenescence: the importance of considering age in health and disease. Clinical and Experimental Immunology 2017, 187: 1–3.

Immune senescence: significance of the stromal microenvironment. Clinical and Experimental Immunology 2017, 187: 6–15.

Innate immune responses in the ageing lung. Clinical and Experimental Immunology 2017, 187: 16–25.

Age‐related alterations in immune responses to West Nile virus infection. Clinical and Experimental Immunology 2017, 187: 26–34.

Intracellular signalling pathways: targets to reverse immunosenescence. Clinical and Experimental Immunology 2017, 187: 35–43.

Ageing and inflammation in patients with HIV infection. Clinical and Experimental Immunology 2017, 187: 44–52.

Considerations for successful cancer immunotherapy in aged hosts. Clinical and Experimental Immunology 2017, 187: 53–63.

Ageing and obesity similarly impair antibody responses. Clinical and Experimental Immunology 2017, 187: 64–70.

The life cycle of a T cell after vaccination – where does immune ageing strike? Clinical and Experimental Immunology 2017, 187: 71–81.

Herpes zoster and the search for an effective vaccine. Clinical and Experimental Immunology 2017, 187: 82–92.

Adult vaccination against tetanus and diphtheria: the European perspective. Clinical and Experimental Immunology 2017, 187: 93–99.

## Convergence of senescence and nutrient sensing in regulating senescent T cell function

Senescent human T cells can be identified by multiple phenotypical and functional parameters, including increased evidence of DNA damage, constitutive activation of p38 mitogen‐activated protein (MAP) kinase, short telomeres, loss of telomerase activity and decreased proliferative activity after activation 1. These cells also lose expression of co‐stimulatory molecules such as CD27 and CD28, but re‐express the naive marker CD45RA and increase during ageing 1. It was shown recently that the decreased proliferative activity and decreased ability to induce telomerase after activation was not a passive response due to cellular dysfunction; instead, this was an actively maintained state that resulted from p38 MAP kinase signalling 2. The inhibition of p38 using a small molecule inhibitor enhanced both the proliferation and telomerase activity of T cells after activation 2. The unexpected observation was that the constitutive p38 activation in senescent human CD4^+^ T cells was induced by a novel mechanism that involved activation of the metabolic sensor 5'‐monophosphate activated protein kinase (AMPK) that is activated by low levels of intracellular adenosine triphosphate (ATP) 3. The senescent T cells also exhibited constitutive AMPK activation. The deprivation of non‐senescent T cells of glucose also induced AMPK and p38 activation, leading to a decrease in both telomerase activity and proliferation of these cells, mimicking the functional phenotype of senescent T cells. This showed an inextricable link between the ageing of T cells and their bioenergetics status. The inhibition of AMPK, p38 or a scaffold molecule transforming growth factor beta (TGF‐β)‐activated kinase binding protein 1 (TAB1), that are found together in a macromolecular complex, enhanced both proliferative activity and telomerase induction in senescent or nutrient‐starved T cells 3. The implications of this include decreased functional activity of T cells in environments that are depleted of nutrients, e.g. in the vicinity of a tumour 4. This may be exacerbated during ageing, as older individuals have increased proportions of senescent cells within the T cell compartment and nutrient deprivation in these cells may exacerbate dysfunctional anti‐tumour activity. Furthermore, a novel way to enhance immunity during ageing may be to modulate the nutrient status of older individuals rather than to target immunity directly 5.

## How do senescent T cells obtain their energy?

Senescent CD8^+^ T cells exhibit mitochondrial dysfunction, increased production of reactive oxygen species (ROS) and impaired mitochondrial biogenesis 6. It was shown that human senescent CD8^+^ T cells utilize glycolysis preferentially to generate ATP, in contrast to the effector memory subset, which is much more metabolically flexible and can either use glycolysis or oxidative phosphorylation (OXPHOS) for functional activities 6. One possibility is that the dependence upon glycolysis results from mitochondrial dysfunction 6. As p38 inhibition enhances proliferative activity of senescent CD8^+^ T cells, it raises the question of how the energy for this enhanced activity is acquired and the implications that this has for mitochondrial function. When p38 signalling was inhibited in senescent human CD8^+^ T cells, autophagy was increased and cleared dysfunctional mitochondria, and this reduced mitochondrial ROS production 6. This occurred by a process involving increased trafficking of the autophagy regulating protein 9 (Atg9) from the endosomes to the lysosomes 6. However, despite the increased mitochondrial fitness, senescent T cells still utilize glycolysis, and not OXPHOS, preferentially for increased proliferation after p38 blockade. Therefore, mitochondrial dysfunction is not the main reason why these cells are dependent upon glycolysis for generating energy. These results reinforce the observations that senescence and nutrient sensing pathways are linked inextricably and can control the function of T cells.

## Killer cell lectin‐like receptor G1 (KLRG1) inhibitory receptor activates AMPK to inhibit natural killer (NK) cell function during ageing

NK cells are a first line of defence during infection and in the response to tumours. NK activity declines during ageing 7, and this may contribute to age‐associated immune decline. A possible reason for decreased NK activity may be through inhibition of the cells by ligation of cell surface inhibitory receptors. One such inhibitory receptor, KLRG1, increases on NK cells in individuals aged >70 years and cells with high KLRG1 express AMPK constitutively 8. Furthermore, KLRG1 activates AMPK by preventing its inactivation by the enzyme PP2C rather than by *de‐novo* activation of AMPK itself. The inhibition of KLRG1 or AMPK enhanced NK cell cytotoxicity, cytokine production, proliferation and telomerase activity. Therefore, inhibitory receptor signalling in NK cells down‐regulate effector function through an AMPK‐dependent process that links nutrient sensing to functional activity in NK cells of older subjects. KLRG1 is also increased in CD8^+^ T cells of older individuals, raising the possibility that a similar process may be involved in these cells. It is not known if this KLRG1/AMPK inhibitory pathway requires p38 MAP kinase for its activation or if this pathway is shared with other inhibitor receptors that can be found on T cells such as programmed death 1 (PD‐1) and cytotoxic T lymphocyte antigen 4 (CTLA‐4). Nevertheless, these observations suggest that inhibitory cell signalling and energy‐sensing pathways converge to inhibit the function of highly differentiated NK as well as T cells. More importantly, this inhibitor pathway can be targeted at different points to enhance functional responses that may be exploited to enhance immunity during ageing.

## Conclusion

The studies outlined above highlight that decreased T and NK cell function in older humans can be regulated by convergent pathways that are initiated by senescence, nutrient deprivation or cell surface inhibitory receptor signalling. Importantly the inhibition of upstream or downstream components of this signalling cascade can enhance both T and NK function. It remains to be determined whether similar mechanisms can also regulate B cell function during ageing. These results suggest collectively that, in future, it may be possible to enhance immune reactivity during ageing by intervention in nutrient‐dependent signalling pathways. These observations also underscore the potential role of dietary intervention to maintain a healthy immune system in older subjects.

## Disclosures

None declared.
